# Tooth Powders. Etc

**Published:** 1869-08

**Authors:** 


### Editorial.

### TOOTH POWDERS, ETC.

S. S. White, ever watchful of the interests of the Dental profession, and neglecting nothing that may minister to its welfare, has, within the last year, made quite an addition to its prophylactic and therapeutic preparations. He has, with the best counsel and advisement, and with the best selection of material, prepared three varieties of Tooth Powder, Nos. 1, 2 and 3, which, from a very thorough test, as well as from a knowledge of the ingredients, we regard as most excellent; we have seen nothing better for general use. It is made of materials superior to those that could be obtained by members of the profession usually, and is far better prepared than it could be in small quantities, and with less perfect facilities. It is furnished to the profession in any quantity from one pound upward.

He has also a dentifrice in the form of a paste. This form makes no special change in quality, but is more a matter of convenience than anything else. This is furnished in mass, or put up in boxes as may be desired.

The profession are now able to obtain these preparations reliable and uniform. T.

